# Genotype-Phenotype Correlations in Charcot-Marie-Tooth Disease Due to *MTMR2* Mutations and Implications in Membrane Trafficking

**DOI:** 10.3389/fnins.2019.00974

**Published:** 2019-10-14

**Authors:** Haicui Wang, Ayşe Kaçar Bayram, Rosanne Sprute, Ozkan Ozdemir, Emily Cooper, Matthias Pergande, Stephanie Efthymiou, Ivana Nedic, Neda Mazaheri, Katharina Stumpfe, Reza Azizi Malamiri, Gholamreza Shariati, Jawaher Zeighami, Nurettin Bayram, Seyed Kianoosh Naghibzadeh, Mohamad Tajik, Mehmet Yaşar, Ahmet Sami Güven, Farah Bibi, Tipu Sultan, Vincenzo Salpietro, Henry Houlden, Hüseyin Per, Hamid Galehdari, Bita Shalbafan, Yalda Jamshidi, Sebahattin Cirak

**Affiliations:** ^1^Department of Pediatrics, University Hospital Cologne, Cologne, Germany; ^2^Center for Molecular Medicine (CMMC), University of Cologne, Cologne, Germany; ^3^Department of Pediatric Neurology, University of Health Sciences, Kayseri City Hospital, Kayseri, Turkey; ^4^Genetics Research Centre, Molecular and Clinical Sciences Institute, St. George's, University of London, London, United Kingdom; ^5^Department of Neuromuscular Disorders, Institute of Neurology, University College London, London, United Kingdom; ^6^Narges Medical Genetics and Prenatal Diagnosis Laboratory, Ahvaz, Iran; ^7^Department of Genetics, Faculty of Science, Shahid Chamran University of Ahvaz, Ahvaz, Iran; ^8^Paediatric Neurology, Department of Paediatric Neurology, Golestan Medical, Educational, and Research Center, Ahvaz Jundishapur University of Medical Sciences, Ahvaz, Iran; ^9^Department of Medical Genetics, Faculty of Medicine, Ahvaz Jundishapur University of Medical Sciences, Ahvaz, Iran; ^10^Department of Ophthalmology, University of Health Sciences, Kayseri City Hospital, Kayseri, Turkey; ^11^Department of Medicine, Azad University, Tehran, Iran; ^12^Department of Neurology, Firoozgar General Hospital, University of Medical Sciences, Tehran, Iran; ^13^Department of Ear Nose and Throat, University of Health Sciences, Kayseri City Hospital, Kayseri, Turkey; ^14^Department of Pediatric Neurology, Meram Medical Faculty, Necmettin Erbakan University, Konya, Turkey; ^15^Institute of Biochemistry and Biotechnology, PMAS Arid Agriculture University, Rawalpindi, Pakistan; ^16^Department of Pediatric Neurology, Institute of Child Health, The Children's Hospital Lahore, Lahore, Pakistan; ^17^Pediatric Neurology and Muscular Diseases Unit, IRCCS Istituto Giannina Gaslini, Genoa, Italy; ^18^Department of Neurosciences, Rehabilitation, Ophthalmology, Genetics, Maternal and Child Health, University of Genoa, Genoa, Italy; ^19^Department of Pediatric Neurology, Erciyes University Medical School, Kayseri, Turkey; ^20^Iran Social Security Organization, Labafinejad Hospital, Tehran, Iran

**Keywords:** Charcot-Marie-Tooth disease type 4B1, myotubularin-related 2 gene, whole-exome sequencing, phosphoinositides, membrane remodeling

## Abstract

Charcot-Marie-Tooth type 4 (CMT4) is an autosomal recessive severe form of neuropathy with genetic heterogeneity. CMT4B1 is caused by mutations in the myotubularin-related 2 (*MTMR2)* gene and as a member of the myotubularin family, the MTMR2 protein is crucial for the modulation of membrane trafficking. To enable future clinical trials, we performed a detailed review of the published cases with *MTMR2* mutations and describe four novel cases identified through whole-exome sequencing (WES). The four unrelated families harbor novel homozygous mutations in *MTMR2* (NM_016156, Family 1: c.1490dupC; p.Phe498IlefsTer2; Family 2: c.1479+1G>A; Family 3: c.1090C>T; p.Arg364Ter; Family 4: c.883C>T; p.Arg295Ter) and present with CMT4B1-related severe early-onset motor and sensory neuropathy, generalized muscle atrophy, facial and bulbar weakness, and pes cavus deformity. The clinical description of the new mutations reported here overlap with previously reported CMT4B1 phenotypes caused by mutations in the phosphatase domain of *MTMR2*, suggesting that nonsense *MTMR2* mutations, which are predicted to result in loss or disruption of the phosphatase domain, are associated with a severe phenotype and loss of independent ambulation by the early twenties. Whereas the few reported missense mutations and also those truncating mutations occurring at the C-terminus after the phosphatase domain cause a rather mild phenotype and patients were still ambulatory above the age 30 years. Charcot-Marie-Tooth neuropathy and Centronuclear Myopathy causing mutations have been shown to occur in proteins involved in membrane remodeling and trafficking pathway mediated by phosphoinositides. Earlier studies have showing the rescue of MTM1 myopathy by MTMR2 overexpression, emphasize the importance of maintaining the phosphoinositides equilibrium and highlight a potential compensatory mechanism amongst members of this pathway. This proved that the regulation of expression of these proteins involved in the membrane remodeling pathway may compensate each other's loss- or gain-of-function mutations by restoring the phosphoinositides equilibrium. This provides a potential therapeutic strategy for neuromuscular diseases resulting from mutations in the membrane remodeling pathway.

## Introduction

Charcot-Marie-Tooth disease (CMT) is a clinically and genetically heterogeneous group of hereditary disorders characterized by chronic degenerative neuropathy of the peripheral nervous system. It is one of the most common causes of peripheral neuropathy affecting at least 1 in every 2,500 people (Szigeti and Lupski, 2009), with more than 80 disease-causing genes being identified (Previtali et al., 2007). CMT4B is a severe autosomal recessive neuropathy with demyelination and myelin outfoldings of the nerve and is classified into two subgroups: CMT4B1 (Bolino et al., 2000) (*MTMR2* mutations, OMIM 601382) and CMT4B2 (*MTMR13* mutations, OMIM 604563). Patients with CMT4B1 have a severe early-onset of disease and are characterized by demyelinating sensorimotor polyneuropathy with irregular redundant loops of focally folded myelin seen in nerve biopsy, called myelin outfoldings (Quattrone et al., 1996).

*MTMR2* encodes the Myotubularin Related Protein 2 (MTMR2) which is part of the large myotubularin family crucially implicated in the modulation of cellular membrane trafficking. In mammals, the myotubularin family has 14 members, MTM1 and MTMR1-13. They all feature a conserved PTP-like (protein tyrosine phosphatase-like) and PH-GRAM (pleckstrin homology- Glucosyltransferases, Rab-like GTPase activators, and Myotubularins) domains (Begley et al., 2003). Among the 13 MTMRs, seven are inactive phosphatases due to substitutions of conserved amino acids for active catalysis, but they can heterodimerize with the active forms for activity regulation (Zou et al., 2012).

MTMR2 is highly expressed in motor and sensory neurons, regulating membrane homeostasis in Schwann cell myelination (Quattrone et al., 1996; Bolino et al., 2000). Limited genotype-phenotype correlations are available for CMT4B1, due to the restricted number of patients carrying *MTMR2* mutations that have been reported to date and lack of scientific interest. Thus, far no specific treatment for CMT4B1 exists.

Both CMT4B1 and Centronuclear myopathy (CNM) are caused by mutations in a set of proteins involved in membrane remodeling, suggesting they may have shared pathological pathways (Cowling et al., 2012), and that there may be some redundancy allowing compensation for the loss of function by restoring the balance of enzyme activities (Raimondi et al., 2011; Raess et al., 2017). Indeed, the rescue of MTM1 myopathies by MTMR2 overexpression has recently been demonstrated (Raess et al., 2017; Danièle et al., 2018).

In this study, we report four different novel *MTMR2* homozygous mutations in four unrelated families with severe clinical presentation, with all four mutations in the phosphatase domain of MTMR2. Through evaluation of the clinical and genetic spectrum of all up to date (beginning of 2019) reported MTMR2 related CMT4B1 cases and correlation between phenotype and genotype, we provide guidance for genetic and clinical diagnosis for *MTMR2*-related CMT4B1.

## Materials and Methods

### Patients

We report four unrelated families from Turkey, Afghanistan, and Iran, comprising of three, three, two and one affected siblings, respectively, all born to consanguineous parents. Patients from family 1 were referred to the University of Health Sciences, Kayseri City Hospital, Department of Pediatric Neurology, Kayseri, Turkey. Patients from family 2 were referred to Dr. Shalbafans private clinic in Ahvaz, Iran. The siblings of family 3 were referred to Narges Medical Genetics and Prenatal Diagnosis Laboratory, Ahvaz, Iran for genetic counseling and diagnosis. A 5-year-old female from family 4 was referred to the Department of Pediatric Neurology, Institute of Child Health and The Children's Hospital Lahore, Pakistan. Consent was obtained individually, and routine diagnosis and related studies were approved by the respective institutional ethical boards in Cologne/Germany, Ahvaz Jundishapur University of Medical Sciences/Iran, the Institute of Child Health and The Children's Hospital Lahore/Pakistan.

### Exome Sequencing

The institutional ethical board of University of Cologne approved the study and informed consent was obtained from the parents. Whole exome sequencing (WES) of an index patient from each family was carried out (experimental WES details for each patient listed in the Supplementary Material).

We used the best-practice filtering scheme based on the autosomal-recessive inheritance from the pedigrees (**Figure 2**) (as previously described in Seelow and Schuelke, 2012; Lupski et al., 2013; Sprute et al., 2019) and evaluated variants using the American College of Medical Genetics and Genomics (ACMG) guidelines and established variant databases for variant interpretation [Mutation Taster (http://www.mutationtaster.org/), gnomAD (http://gnomad.broadinstitute.org/gene/), ClinVar (https://www.ncbi.nlm.nih.gov/clinvar/), GME (http://igm.ucsd.edu/gme/data-browser.php), and ExAc (http://exac.broadinstitute.org)]. The mutations were further evaluated with established prediction tools MutPred2, SIFT, PolyPhen, and PhyloP scores (Richards et al., 2015). Dideoxy sequencing was performed for confirmation and cosegregation of variants in available family members.

### Pathway Analysis

We used Cytoscape stringapp for StringDB interaction network analysis (von Mering et al., 2005; Doncheva et al., 2019). The String database consists of PPI information from experimental data, functional associations from curated pathways, text mining from publications and predictions. The probabilistic scores from each functional association are combined with a confidence score for each PPI and corrected for randomly observed interactions. Seven neuromuscular disease-associated genes involved in membrane remodeling (*n* = 7) were selected for the analysis (**Figure 4**). The top 20 interactors with the highest confidence scores were added to the dataset. Note that all analysis details including the enriched terms from different databases as well as PIMDs (interaction evidence), PPI interaction between every 2 nodes, related *p*-values, and genes included for each enriched term are exported in the spreadsheet tables (Supplementary Table 1 EdgeTable and Supplementary Table 2 StringTable). The network maps represent a short summary of these tables: the colored donut slices which surround each node represent the most significant eight interactions of the enriched data in which that gene (node) was found. The color codes can also be found in the Supplementary Table 2 String Table. The over-representation analysis (ORA) was performed for Gene Ontology (GO) Cellular Component (GO:CC) (Supplementary Table 3), Biological Process (GO:BP, Supplementary Table 4), Molecular Function (GO:MF, Supplementary Table 5) classes (Boyle et al., 2004) via R-cluster profile library (Yu et al., 2012). Hypergeometric distribution was used to calculate the *p*-values. False discovery rates (FDR) were calculated via the Benjamini-Hochberg method. A total of 27 genes (7 selected neuromuscular disease-associated genes in addition to 20 the most significant genes according to interactome analysis). The FDR adjusted *p*-values below 0.05 accepted as statistically significant GO terms. More details on the ORA can be found in the Supplementary Material.

## Results

### Clinical Description

#### Family 1

**Figure 1:**
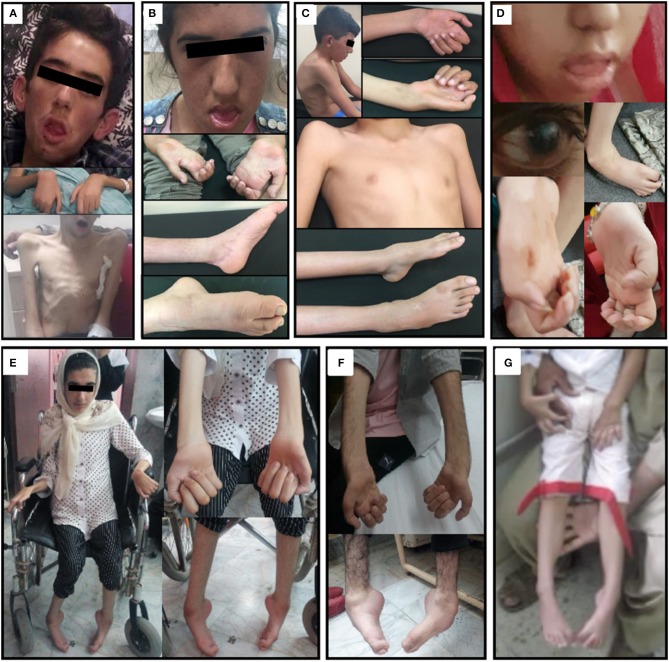
Clinical hallmarks. **(A)** The clinical picture of patient family 1 II.1 at the age of 18 years showing generalized muscle atrophy, facial weakness, and mild pectus carinatum. **(B)** Clinical presentation of patient family 1 II.2 at the age of 17 years with facial weakness, progressive neuropathic muscular atrophy in the hands and pes cavus foot deformities. **(C)** Patient family 1 II.3 at the age of 12 years presenting with thoracic kyphosis, progressive neuropathic muscular atrophy in the hands, mild pectus carinatum and pes cavus foot deformities. **(D)** Clinical presentation of patient II.3 from family 2 showing facial weakness, clenched hands, club feet, and glaucoma. **(E,F)** Clinical hallmarks from the affected individuals in Family 3 illustrate the skeletal deformities. **(E)** Distal muscle atrophy, claw hand deformities, hammertoes, and foot deformities in the wheelchair-bound bound girl (Family 3, Patient II.2). **(F)** Distal muscle atrophy, claw hand deformities, and foot drop in the elder brother (Family 3, Patient II.1). **(G)** Clinical presentation of patient family 4 II.3 showed pes cavus foot deformity and muscle weakness and atrophy mainly involving the lower extremity muscles.

Three Turkish siblings, one female and two males (Figures 1A–C), were referred to our clinic with complaints of severe generalized muscle weakness and motor developmental delay. All patients were born at term after an uneventful pregnancy via uncomplicated vaginal delivery without having any postnatal problems. Their parents were first-degree consanguineous, having three affected children among a total of nine. All three patients developed symptoms with slow progression after reaching the age of 3 years (see Supplementary Materials for detailed clinical description and Supplementary Videos 1–3) (Neyzi et al., 2015). Their tendon reflexes were absent in upper and lower extremities, without any pathological reflexes (Medical Research Council, 1976). Biochemical tests including a complete blood count, liver and kidney function tests, serum electrolyte levels, tests for common inborn errors of metabolism, thyroid function tests, and muscle creatine kinase levels revealed normal values. Brain and spine MRI results were not pathological. Electrophysiological studies showed severe demyelinating sensorimotor polyneuropathy (Tables 1, 2).

**Table 1:** Clinical, electrophysiological, and molecular genetic characteristics of the sibling patients from family 1, 2, 3, and 4.

**Findings**	**Family 1 patient II.1**	**Family 1 patient II.2**	**Family 1 patient II.3**	**Family 2 patient II.1**	**Family 2 patient II.3**	**Family 2 patient II.4**	**Family 3 patient II.1**	**Family 3 patient II.2**	**Family 4 patient II.3**
Age/Gender	18 years^†^/Male	18 years/Female	12 years/Male	29 years/Female	26 years/Female	24 years/Male	20 years/Male	18 years/Female	5 years/Female
Age of symptom onset	4 years	4 years	4 years	7 years	1.5 years	6 years	4 years	5 years	First months of life
Growth development	Height and Weight <3rd percentile	Height and Weight <3rd percentile	Height and Weight <3rd percentile	Height and Weight <3rd percentile	Height and Weight <3rd percentile	Height and weight normal	Height and Weight <3rd percentile	Height and Weight <3rd percentile	Not elicited
Muscles strength (MRC grade)	Upper limbs: 3/5 in proximal, and 2/5 in distal Lower limbs: 0/5 in proximal and distal	Upper limbs: 4/5 in proximal, and 3/5 in distal Lower limbs: 4/5 in proximal, and 3/5 in distal	Upper limbs: 5/5 in proximal, and 4/5 in distal Lower limbs: 5/5 in proximal; 4/5 in the left distal, and 3/5 in the right distal	Upper limbs: 5/5 in proximal, and 4/5 in distal Lower limbs: 5/5 in proximal; 4/5 in the left distal, and 3/5 in the right distal	Upper limbs: 4/5 in proximal, and 0/5 in distal Lower limbs: 0/5 in proximal; 0/5 in distal	Upper limbs: 5/5 in proximal, and 5/5 in distal Lower limbs: 5/5 in proximal; 2/5 in distal	Not elicited	Not elicited	Not elicited
DTRs	Absent	Absent	Absent	Absent	Absent	Absent	Absent	Absent	Absent
Gait	Wheelchair-bound at age 17	Wheelchair-bound at age 18	Walk without support	Wheelchair-bound at age 15	Wheelchair-bound at age 15	Walk without support	Steppage gait	Wheelchair-bound	Walk without support
**Electrophysical findings**									
Ulnar MAP at wrist (m/V)	Not elicited	Not elicited	1.1	Absent response	Absent response	Absent response	Not elicited	Not elicited	Not elicited
Ulnar MCV at wrist (m/s)	Not elicited	Not elicited	8.15	Absent response	Absent response	Absent response	Not elicited	Not elicited	Not elicited
Ulnar SNAP at wrist (μV)	Absent response	Absent response	Absent response	9.6 (norm>15)	Absent response	Absent response	Not elicited	Not elicited	Not elicited
Ulnar SCV at wrist (m/s)	Absent response	Absent response	Absent response	34 (norm>38)	Absent response	Absent response	Not elicited	Not elicited	Not elicited
Ulnar MAP below elbow (m/V)	Not elicited	Not elicited	0.6	Absent response	Absent response	Absent response	Not elicited	Not elicited	Not elicited
Ulnar MCV below elbow (m/s)	Not elicited	Not elicited	20.2	Absent response	Absent response	Absent response	Not elicited	Not elicited	Not elicited
Ulnar SNAP below elbow (μV)	Absent response	Absent response	Absent response	8.5 (norm>10)	Absent response	Absent response	Not elicited	Not elicited	Not elicited
Ulnar SCV below elbow (m/s)	Absent response	Absent response	Absent response	33 (norm>39)	Absent response	Absent response	Not elicited	Not elicited	Not elicited
Tibial MAP (mV)	Not elicited	Not elicited	1.2	Not elicited	Not elicited	Not elicited	Not elicited	Not elicited	Not elicited
Tibial MCV (m/s)	Not elicited	Not elicited	8.95	Not elicited	Not elicited	Not elicited	Not elicited	Not elicited	Not elicited
Tibial SNAP (μV)	Absent response	Absent response	Absent response	Not elicited	Not elicited	Not elicited	Not elicited	Not elicited	Not elicited
Tibial SCV (m/s)	Absent response	Absent response	Absent response	Not elicited	Not elicited	Not elicited	Not elicited	Not elicited	Not elicited

† *Passed away; DL, distal latency; DTR, deep tendon reflex; MAP, compound muscle action potential; MCV, motor conduction velocity; MRC, Medical Research Council Grades for Muscle Strength; SNAP, sensory nerve action potential; SCV, sensory conduction velocity*.

**Table 2:** Overview of MTMR2 related Charcot-Marie-Tooth disease type 4B1.

**References**	**Age/Gender (M/F)**	**Age at onset (months)**	**Nerve palsy**	**Skeletal deformities**	**Systemic problems**	**Demyelinating PNP**	**MTMR2 mutation**
Houlden et al., 2001	16 years/M	13	Vocal	No	Stridor, Dysarthria	Yes	c.308G>A; p.Gly103Glu
	5 years/M	12	No	No	No	No	c.324del; p.Asp109IlefsTer17
Nelis et al., 2002	10 years/M	24	No	No	No	Yes	c.847C>T; p.Arg283Trp
	16 years/F	24	No	No	No	No	c.847C>T; p.Arg283Trp
Verny et al., 2004	18 years/F	Birth	No	Pes equinovarus	Dysarthria, respiratory difficulties	Yes	c.1749G>A; p.Trp583Ter
Parman et al., 2004	21 years/F	Delayed motor milestones	No	Pes cavus, claw hands, scoliosis	Hypophonia	Yes	c.681_682ins(446); p.Thr228fsTer275
	24 years/F		No	Pes cavus, hammer toe	Hypophonia, tremor	Yes	c.841_844del; p.Ile281LeufsTer10
Nouioua et al., 2011	16 years/M	24	Vocal, facial	Pes equinovarus, claw hands, chest	Stridor	Yes	c.331dup; p.Arg111LysfsTer24
	12 years/M	24	Vocal, facial	Claw hands, chest	Stridor	Yes	c.331dup; p.Arg111LysfsTer24
	9 years/M	12	Vocal, facial	Pes equinovarus, claw hands, chest	Stridor	Yes	c.331dup; p.Arg111LysfsTer24
Luigetti et al., 2013	48 years/M	No data	No	No	Facial weakness	No	c.1534del; p.Leu512TyrfsTer33
	30 years/F	No data	No	No	Respiratory failure	Yes	c.1534del; p.Leu512TyrfsTer33
Murakami et al., 2013	30 years/F	156	No	Claw hands	No	Yes	c.1882_1885del; p.Arg628ProfsTer18
Scott et al., 2016	28 years/M	48	No	Claw hands	Facial weakness, optic neuritis, cervical Schwannoma	Yes	c.1768C>T; p.Gln590Ter
Zambon et al., 2017	3 years/F	12–18	Vocal	Pes planus, foot-drop	Facial weakness, hoarseness, stridor, dysphagia	Yes	c.484 C>T; p.Arg162Ter
Abdalla-Moady et al., 2018	31 years/F	132	No	Pes cavus	No	No data	c.1877_1878insAGAG, p.Ala629GlufsTer31
Current report: Family I 3 siblings	18 years/M	48	Vocal	Pes cavus, mild pectus carinatum, thoracic kyphosis	Facial weakness, dysphagia, speech impairment, adenoid hypertrophy, arytenoid subluxation, myopic refractive error	Yes	c.1490dupC; p.Phe498IlefsTer2
	17 years/F	48	Vocal	Pes cavus, mild pectus carinatum	Facial weakness, dysphagia, speech impairment, adenoid hypertrophy, arytenoid subluxation, myopic refractive error	Yes	c.1490dupC; p.Phe498IlefsTer2
	12 years/M	48	No	Pes cavus, mild pectus carinatum, thoracic kyphosis	Facial weakness, speech impairment, adenoid hypertrophy, hypoplastic arytenoid cartilages with medial deviation	Yes	c.1490dupC; p.Phe498IlefsTer2
Current report Family II 3 siblings	29 years/F	84	No	Pes cavus, pectus carinatum, thoracic kyphosis	Facial weakness, severe dysphonia dysphagia, respiratory attacks	Yes	Splice site c.1479+1G>A
	26 years/F	18	No	Pes cavus, club feet, clenched hands	Facial weakness, bulbar muscle weakness, bilateral glaucoma with loss of vision	Yes	Splice site c.1479+1G>A
	24 years/M	72	No	Pes cavus	Mild facial weakness, moderate dysphonia, seizures	Yes	Splice site c.1479+1G>A
Current report Family III 2 siblings	20 years/M	48	No	Pes cavus, mild scoliosis, hammer toes, foot drop, claw hands	Dysphonia	Yes	c.1090C>T; p.Arg364Ter
	18 years/F	60	No	Pes cavus, severe scoliosis, hammer toes, foot drop, claw hands	Facial weakness	No data	c.1090C>T; p.Arg364Ter
Current report Family IV 1 sibling	5 years/F	First months of life	No	Pes cavus		Yes	c.883C>T; p.Arg295Ter

*F, female; M, male; PNP, Polyneuropathy; EMG, Electromyography.*

*A PubMed/Medline search (1966–2019) of “Charcot-Marie-Tooth disease AND MTMR2 mutation” and “Charcot-Marie-Tooth disease type 4B1.” Cohort of Pareyson et al. was not included due to overlapping of the reviewed cases and the absence of a clear phenotypic assignment of the novel cases. MTMR2 reference sequence (NM_016156)*.

#### Family 2

Three Afghani siblings, one male and two females (Figure 1D and Supplementary Figures 1A,B), presented with complaints of severe generalized muscle weakness. Their parents were first-degree relatives, with three affected and three unaffected children. All patients were born at term after an uneventful pregnancy via uncomplicated vaginal delivery without any postnatal problems. Their tendon reflexes were absent in upper and lower extremities, without any pathological reflexes (see Supplementary Materials for detailed clinical description and Supplementary Figure 1). Biochemical tests including a common test for inborn errors of metabolism, thyroid functions, and muscle creatine kinase levels revealed values in the normal range. Brain and spine MRI results were normal. Electrophysiological studies showed severe demyelinating sensorimotor polyneuropathy (Table 1 and Supplementary Figure 2).

#### Family 3

Two siblings, one female, and one male, were born to a consanguineous family in Iran, who presented with skeletal defects and motor disabilities. Both patients were born via normal vaginal delivery and appeared normal at birth without any remarkable complaints.

Patient II.2 from family 3 is a 20-year-old male. He presented with delayed walking at the age of 2 years. From the age of four, he began to progressively show signs of neuropathic symptoms including muscle weakness, foot drop, pes cavus, hammertoes, and claw hand deformities. He was exhibiting a steppage gait (Figure 1F and Supplementary Video 4). The electrophysiological studies at 5–6 years were compatible with a demyelinating sensorimotor neuropathy. The latest clinical exam confirmed that he developed distal and proximal limb muscle atrophy, mild scoliosis, and an abnormal voice that reflects the involvement of laryngeal muscles. However, his cognition was normal and he had no facial weakness. Patient II.1 from family 3 is a wheelchair-bound 18-year old female (Figure 1E) who presented with peripheral nervous system and skeletal abnormalities and had a symptom onset at 5 years. The latest neurological examination demonstrated that specific symptoms and disease progression were similar to her older brother (Table 1), although the severity of the disease was much more serious compared to him. Furthermore, she has shown facial weakness.

#### Family 4

Patient II. 3 from family 4 is a 5-year-old female with a history of weakness and motor developmental delay (including delay in sitting and crawling) from the first months of life and a pes cavus foot deformity. Her healthy parents are Pakistani second-degree cousins from the region of Punjab. She started walking at the age of 2 years. Her presentation was resembling a motor polyneuropathy with progressive deterioration. Neurological examination revealed a pes cavus foot deformity and muscle weakness and atrophy mainly involving the lower limb muscles (Figure 1G). Deep tendon reflexes were absent. Her healthy parents were Pakistani second-degree cousins from the region of Punjab.

### Genomic Results

**Figure 2:**
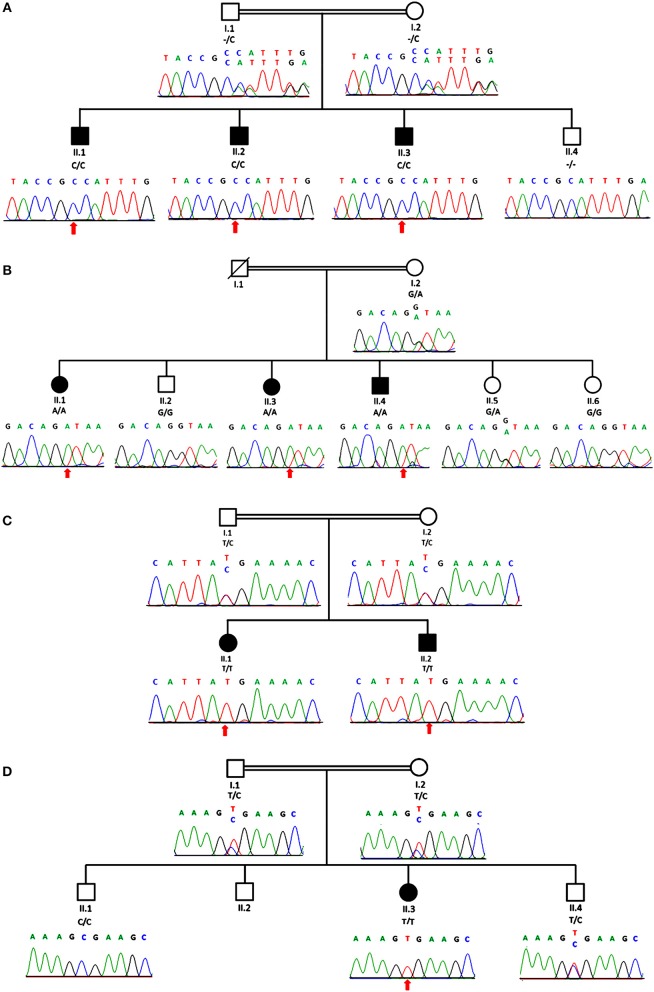
Genomic analysis revealed an individual mutation in *MTMR2* gene. The genealogy of the family and the *MTMR2* electropherograms show the same novel homozygous c.1490dupC; p.Phe498IlefsTer2 mutation identified in all three siblings in family 1 **(A)** and a homozygous c.1479+1G>A splice site mutation in family 2 **(B)** and in family 3 **(C)** a homozygous c.1090C>T; p.Arg364Ter stop mutation and in family 4 **(D)** a homozygous c.883C>T; p.Arg295Ter mutation in the *MTMR2* gene was revealed. Parents were heterozygous for the same mutation as their children in all three families.

Exome sequencing revealed a homozygous c.1490dupC; p.Phe498IlefsTer2 mutation in *MTMR2* (NM_016156) in the index patient of family 1, that was found to co-segregate; a homozygous splice site mutation c.1479+1G>A in *MTMR2* co-segregating in family 2; a homozygous nonsense mutation c.1090C>T; p.Arg364Ter in *MTMR2* co-segregating in family 3 which lies in an extended region of homozygosity that spans 52.47 Mb; and a homozygous nonsense mutation in *MTMR2* (c.883C>T; p.Arg295Ter) co-segregating in family 4. These variants have not been previously described nor published elsewhere to be associated with CMT4B1 and were confirmed via dideoxy sequencing to be co-segregating with the phenotype in the four families (Figure 2).

In family 1, both parents were confirmed to be heterozygous carriers for the same mutation. For family 2, a DNA sample of the father was not available, but the mother carries the same heterozygous variant as her affected children do in the homozygous state. The parents in family 3 and 4 were also confirmed to be heterozygous carriers for the same mutation as their children.

Three of four novel mutations from our study are predicted to result in non-sense mediated decay, the c.1490dupC; c.1090C>T; c.883C>T. Following NMD exclusion guidelines suggested by Popp and Maquat (2016): the premature stop codon should be located 50–55 or fewer nucleotides upstream of an exon-exon junction to prevent NMD. That is not the case for these three mutations, whose stop codon location did not meet the criteria to prevent NMD, thus they are very likely to be degraded by NMD. However, this prediction still requires experimental validation.

## Discussion

**Figure 3:**
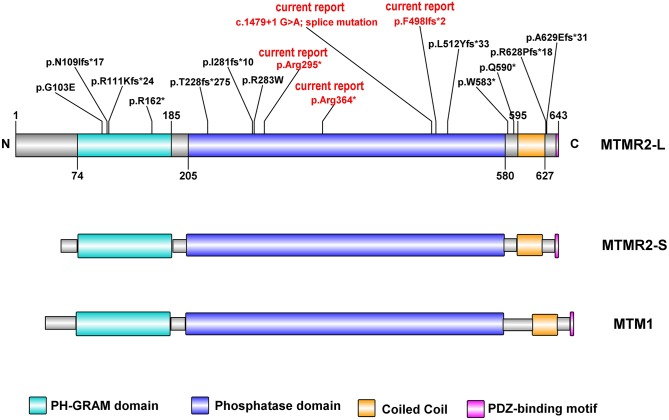
Schematic representation of human MTMR2 protein domains (NP 057240.3) and position of the mutations identified so far, generated by Illustrator for Biological Sequences: IBS. In red are the reported novel mutations identified in our study. The MTMR2 long isoform (MTMR2-L), MTMR2 short isoform (MTMR2-S), and MTM1, all contain (Raess et al., 2017) PH-GRAM domain, phosphatase domain, Coiled-Coil domain, and PDZ binding motif. The two MTMR2 protein isoforms differ only in their translation start sites.

**Figure 4:**
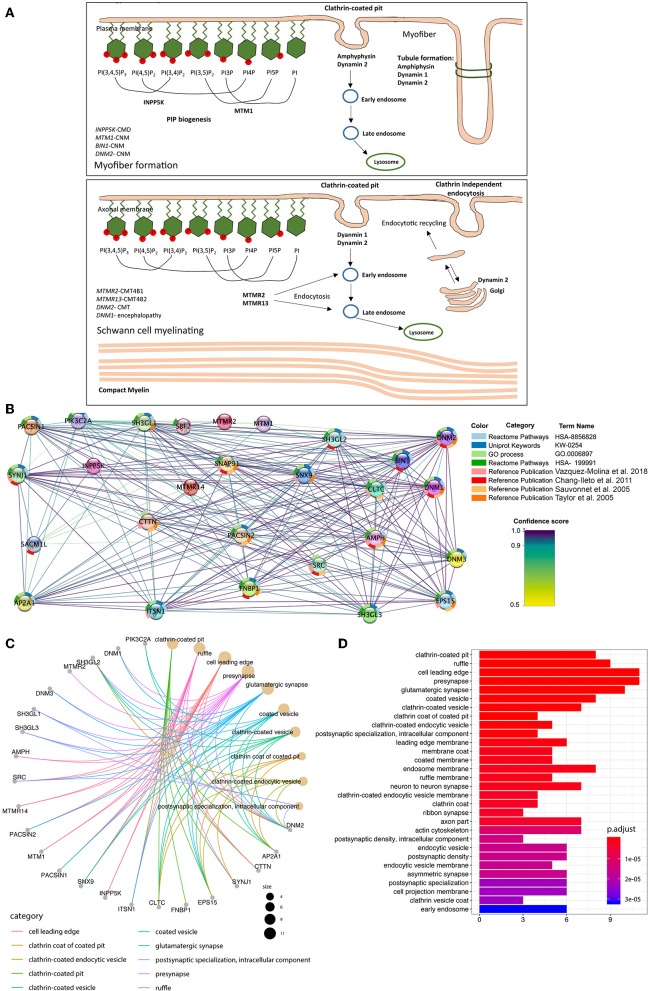
Illustration of neuromuscular disease-associated proteins involved in membrane remodeling. A high resolution figure can be found and downloaded together with the Supplementary Material file. **(A)** The summarized known genes involved in both myopathy and neuropathy, which encode essential proteins for phosphoinositides equilibrium, membrane remodeling and trafficking based on previous studies (Berger et al., 2006; Cowling et al., 2012). Proteins are classified to groups based on the steps they are involved in membrane remodeling, and related diseases are also listed. **(B)** GO over-representation analysis with input genes (*n* = 7) are selected from **(A)**. Then 20 additional interactors added to the dataset, respectively according to their combined confidence score. Protein-Protein interaction (PPI) information visualized with Cytoscape-string app. The network map shows 27 PPI which has the highest scores for the given set of genes. In the map proteins are represented via nodes; the confidence scores are represented via interaction line colors; the enrichment terms are represented with donut slices surrounding each node. **(C)** A total of 27 genes which have found to be the most significant in the StringDB analysis was used for the over-representation analysis. First, 30 Gene-ontology Cellular Component terms enriched in the analysis are shown in **(A)**. The colors represent the FDR adjusted *p*-values for each term. **(D)** The cnet plot shows the linkage between genes and first 10 Gene Ontology Cellular Component (GO:CC) terms in which those genes are over-represented. The size of GO term nodes represents the count of genes which are over-represented in that term.

Myotubularin (MTM1) and MTM related proteins (MTMRs) belong to the large subfamily of protein tyrosine phosphatases (PTPs). Mutations in *MTMR2* underlie autosomal recessive CMT4B1. MTMR2 consists of a PH-GRAM (pleckstrin homology- Glucosyltransferases, Rab-like GTPase activators and Myotubularins) domain, a phosphatase domain, one coiled-coil and a PDZ-binding motif (Begley et al., 2003; Figure 3). It has four isoforms in mice. The longest isoform (MTMR2-L) has a longer N-terminal sequence before the PH-GRAM domain compared to the shortest one (MTMR2-S). MTMR2 can dephosphorylate both Phosphatidylinositol 3-phosphate (PtdIns3P) and Phosphatidylinositol 3,5-bisphosphate [PtdIns(3,5)P_2_; (Begley et al., 2003)], which are regulators of membrane homeostasis and vesicle transport. Disruption of PtdIns(3,5)P_2_ homeostasis, which is maintained by two phosphatases MTMR2 and FIG4 (Factor-Induced Gene 4), is linked to altered longitudinal myelin growth and myelin outfolding formation and is believed to be the mechanism for *MTMR2* mutations causing CMT4B1 (Vaccari et al., 2011).

CMT4B1 is characterized by severe progressive distal and proximal muscular weakness in early childhood, initially starting at the lower limbs, as well as sensory loss and decreased nerve conduction velocity (Quattrone et al., 1996; Bolino et al., 2000; Houlden et al., 2001; Parman et al., 2004; Verny et al., 2004; Nouioua et al., 2011; Scott et al., 2016). We report nine children from four unrelated families with a typical CMT4B1 phenotype of severe early-onset motor and sensory neuropathy. Distal muscle weakness was distinct in all our patients. Sensory nerve action potentials and nerve conduction velocity could not be detected in any of the cases of family 1. Compound muscle action potential and motor conduction velocity could be measured only in patient II.3 from family 1 at severely reduced levels (Table 1). In agreement with most previously reported cases of CMT4B1 (Bolino et al., 2000; Houlden et al., 2001; Parman et al., 2004; Verny et al., 2004; Nouioua et al., 2011; Murakami et al., 2013; Scott et al., 2016), our cases were also sired by consanguineous parents.

As the disease progresses, patients usually lose mobility and become wheelchair-bound by the second or third decade of their lives. One of our patients (family 1 II.1) died at the age of 18 years, and three others had a severe motor impairment and were wheelchair-bound since the age of 15 years. Pes cavus deformity was frequently reported in previous studies (Parman et al., 2004; Verny et al., 2004; Nouioua et al., 2011). In the present study, pes cavus deformity was diagnosed in all nine patients and became progressively worse, frequently requiring surgery in the first decade of their lives. Other associated clinical features are developmental delay, facial weakness, claw hands, chest deformity, kyphosis, scoliosis, facial palsy, deafness, schwannoma, optic neuritis, vocal cord palsy, dysarthria, dysphagia, hypophonia, stridor, and respiratory insufficiency (compare Tables 2, 3) (Quattrone et al., 1996; Bolino et al., 2000; Houlden et al., 2001; Nelis et al., 2002; Parman et al., 2004; Verny et al., 2004; Nouioua et al., 2011; Luigetti et al., 2013; Murakami et al., 2013; Scott et al., 2016; Zambon et al., 2017; Abdalla-Moady et al., 2018).

**Table 3:** Clinical phenotype of patients with MTMR2 related Charcot-Marie-Tooth disease.

**Clinical findings**	**Frequency**
**Skeletal deformities**	
Chest deformities	7/25 (28%)
Thoracic kyphosis	3/25 (12%)
Scoliosis	3/25 (12%)
**Hand deformities**	
Claw hands	8/25 (32%)
Clenched hands	1/25 (4%)
**Foot deformities**	
Pes cavus	12/25 (48%)
Pes equinovarus	3/25 (12%)
Pes planus	1/25 (4%)
**Bulbar/laryngeal findings**	
Facial palsy	3/25 (12%)
Facial weakness	10/25 (40%)
Dysphagia	5/25 (20%)
Vocal palsy	7/25 (28%)
Respiratory difficulties	8/25 (32%)
Stridor	5/25 (20%)
Speech impairment	11/25 (44%)
Adenoid hypertrophy	3/25 (12%)
Arytenoid subluxation	2/25 (8%)
Hypoplastic arytenoid	1/25 (4%)
**Eye disease**	4/25 (16%)
Myopic refractive error	2/25 (8%)
Optic neuritis	1/25 (4%)
Glaucoma	1/25 (4%)
**Further clinical findings**	
Cervical schwannoma	1/25 (4%)
Seizures	1/25 (4%)

In family 1, the frameshift *MTMR2* mutation p.Phe498IlefsTer2 is located in the protein tyrosine phosphatase domain and causes a severe form of CMT4B1. All affected family members developed muscle atrophy and facial weakness at the end of the first decade. During the follow-up period, patients II.1 and II.2 from family 1 developed dysphagia and all three siblings had a speech impairment. Additionally, snoring was observed in patient II.1, probably due to adenoid vegetation or arytenoid subluxation and vocal cord paralysis. These two patients represent the first CMT4B1 cases with protein tyrosine phosphatase domain mutations and vocal cord paralysis. The mutation in this family is predicted to result in partial loss of the phosphatase domain (impact on activity) and loss of the entire C-terminal region including the coiled-coil for dimerization (further impact on activity) and membrane association as well as the PDZ binding motif, most likely leading to the severity of the phenotype.

Interestingly, there were hypoplastic arytenoid cartilages with medial deviation and arytenoid subluxation in the patients from family 1. Our findings suggest that arytenoid abnormalities whether or not coupled with vocal cord paralysis due to unknown causes may be associated with the hereditary neuropathy. Vocal cord paralysis has previously been reported with CMT4B1 (Houlden et al., 2001; Nouioua et al., 2011; Zambon et al., 2017). All patients suffered from severe demyelinating neuropathy with respiratory problems. In the present study, vocal cord paralysis developed in patients II.1 and II.2 from family 1. It presented as a late symptom in contrast to previously reported cases (Nouioua et al., 2011; Zambon et al., 2017). Adenoidal vegetation and myopic refractive error are common problems in childhood. The coexistence of these disorders and CMT4B1 may be coincidental in our patients. On the other hand, tonsillitis and adenoidal vegetation must be followed closely to prevent upper airway obstruction in CMT4B1 patients, particularly if they have vocal cord paralysis. It should not be forgotten that in such a clinical condition a mild upper airway infection might have lethal consequences.

In family 2, the homozygous splice-site mutation likewise causes a severe phenotype in the affected siblings. All children developed walking difficulties within their first decade of life and two of them became wheelchair-bound at the age of 15 years. Interestingly, their younger brother (case II.4 of family 2) had a milder clinical course with similar age of onset but a less progressive course, despite having the identical homozygous pathogenic variant as his siblings. With 24 years, he is still able to walk without support, he has no respiratory difficulties and no dysphagia, and his weight and height are within the reference range. On the other hand, he suffered from seizures in his first decade of life. Furthermore, one of the sisters (II.2 of family 2) developed loss of vision due to bilateral glaucoma, a symptom that was not described yet in CMT4B1 patients but has been seen in CMT4 subtype B2 which is caused by mutations in MTMR13 (Pareyson et al., 2019). Nevertheless, it should be noted, that without evidence from additional cases association of CMT4B1 with glaucoma could be a consequence of additional pathogenic variants in a different gene harbored by this affected family member, particularly given the consanguinity.

Thus, we demonstrate in family 2 intra-familial phenotypic variability in regard to the disease course and severity, implicating other factors such as access to high-quality medical care (e.g., environmental modifications, modifier genes, epigenetic alterations) in the clinical outcomes. This variability highlights a promising area of future research aimed at understanding the role of modulatory and environmental factors which contribute to the phenotypes. Due to the limited access to high-quality medical care these families represent the unmitigated natural disease course of the disease.

In family 3, all patients carry a nonsense mutation p.Arg364Ter predicted to result in a truncated protein with loss of substantial part of the phosphatase domain, causing also a severe phenotype in the affected siblings. Both affected siblings developed muscle atrophy at the end of their first decade. In this family, we can also see a phenotypic variability in the symptoms and differences in the disease course despite both siblings harboring the identical pathogenic variant. The older brother is still able to walk at the age of 20 years while his younger sister is already wheelchair bound and is also suffering from facial weakness. Nevertheless, the age of onset was similar to previously reported cases and both children are suffering from muscle weakness, skeletal deformities, and early onset sensorimotor neuropathy, thus exhibiting typical symptoms of the *MTMR2*-related disease.

Interestingly, in both families 2 and 3 the female siblings seem to suffer from a more severe disease course than their male siblings with the same disease-causing mutation.

Similar to family 3, the patient II.3 of family 4 carries a nonsense mutation, p.Arg295Ter predicted to result in a truncated protein with loss of major part of the phosphatase domain, thus also associated with a severe phenotype. The patient exhibited first symptoms of the disease even earlier than reported in the other cases, within the first months of her life. The disease course is not yet comparable due to the young age of the patient, but she already has motor developmental delay and pes cavus deformity which developed by the age of 5 years.

In the previously reported cases (Table 2); the mean age at the time of diagnosis was 3.5 ± 3.7 years (range: 0–13 years). All cases presented with motor and sensory polyneuropathy with different clinical features and progression. So far, four mutations occur in the PH-GRAM domain, five mutations occur in the phosphatase domain, two in the linker area between the phosphatase domain and the following coiled-coil, two in the PDZ-binding motif (Figure 3). Among the four PH-GRAM domain mutations, three of them (p.Asn109IlefsTer17, p.Arg111LysfsTer24, p.Arg162Ter) are predicted to result in a truncated form of MTMR2 lacking the entire phosphatase domain, and except for the patient carrying p.Asn109IlefsTer17 with mild phenotype who is still young (5 years old), the other two already have a severe phenotype (facial weakness, stridor, dysphagia, skeletal deformities, vocal cord paralysis, and facial nerve palsy) corresponding to the predicted loss of enzymatic function of MTMR2. Molecular analysis of all previously reported cases of CMT4B1 with the presentation of vocal cord paralysis has been associated with mutations in the PH-GRAM domain (Houlden et al., 2001; Nouioua et al., 2011; Zambon et al., 2017). Among the mutations occurring in the phosphatase domain, p.Thr228fsTer275 resulted in truncated MTMR2 with loss of the majority of the phosphatase domain including active site residues of PtdIns (3,5)P_2_ binding (Asn330, Asn355, Cys418, Arg423, Arg459, Arg463) (Begley et al., 2006) and is associated with a rather severe phenotype (delayed motor milestones, hypophonia, and skeletal deformities) but less severe than seen in association with truncation of the PH-GRAM domain. This is similar to the severe phenotype observed for patient IV.2 from family 3 which harbors the p.Arg364Ter mutation, predicted to result in loss of >50% of the phosphatase domain including several active site residues of PtdIns(3,5)P2 binding (Cys418, Arg423, Arg459, Arg463). The milder phenotype observed in the brother might be due to variable genomic background. The other two novel mutations described in the current report, p.Phe498IlefsTer2 in family 1, and the splice site mutation c.1479+1G>A in family 2 as well as one previously reported mutant p.Leu512TyrfsTer33 (Luigetti et al., 2013) occur in the tail of the phosphatase domain, presumably resulting in less disruption of the enzymatic function of MTMR2, however together with the predicted complete loss of the C terminus including the coiled-coil domain which is important for dimerization of MTMR2, patients still exhibit a severe phenotype. Two mutations in the coiled-coil domain (p.Gln590Ter, p.Trp583Ter) have also been linked to a rather severe phenotype. This might be due to disruption of the coiled-coil domain which is important for dimerization of MTMR2 and also membrane association (Berger et al., 2003). Mutations located after the coiled-coil domain may retain the partial function of MTMR2 because CMT4B1 cases with *MTMR2* mutations in this localization have a mild phenotype. It was experimentally confirmed that p.Arg628ProfsTer18 protein expression was still persisting as shown by Murakami et al., a faint staining was also observed in the Schwann cells of both control and patient sural nerve biopsy in MTMR2 immunoreactivity (Murakami et al., 2013). This suggested that the disease-causing mechanism of p.Arg628ProfsTer18 might be due to the loss of the PDZ-binding motif, which is a motif important for protein-protein interaction and whose exact role in protein MTMR2 remains unclear. The mutant p.Gly103Glu in the PH-GRAM domain and p.Arg283Trp in the phosphatase domain with only single amino acid change resulted in rather a mild phenotype (the patients are able to walk unassisted with lightweight orthoses) suggesting MTMR2 might retain some low level of activity. However, it is reported that p.Gly103Glu has an impaired membrane association (Berger et al., 2003), while p.Arg283Trp remains mainly as a monomer (Goryunov et al., 2008) as dimerization is required for phosphorylation activity, thus these two mutants still manifest as CMT4B1. Nevertheless, the systematic experimental evaluation of the mutations on protein expression, stability, and enzymatic activity is required for a better understanding of the genotype-phenotype correlations.

Thus, so far, several proteins involved in membrane remodeling have been reported to be associated with neuromuscular diseases. Among these, mutations in myotubularin (*MTM1*), amphiphysin 2 (*BIN1*), and dynamin 2 (*DNM2*) lead to different forms of centronuclear myopathy (CNM). Myotubularins maintain the equilibrium of phosphoinositides, amphiphysin senses the membrane curvature and membrane remodeling while the dynamin 2 as GTPase tubulate and cleave membranes. This suggests that CNM is caused by mutations along a common pathological pathway of membrane remodeling (Cowling et al., 2012). Importantly, MTMRs also belong to the myotubularin family with highly conserved domains (Figure 3), and mutations in MTMRs cause Charcot-Marie-Tooth neuropathies. More interestingly, mutations in dynamin 2 can also cause a dominant form of Charcot-Marie-Tooth neuropathy, and recently it was reported that mutations in another dynamin family member, dynamin 1, cause encephalopathy (von Spiczak et al., 2017). Moreover, mutations in *INPP5K*, which encodes the inositol polyphosphate-5-phosphatase K, also known as SKIP (skeletal muscle and kidney enriched inositol phosphatase), cause congenital muscular dystrophy (Osborn et al., 2017; Wiessner et al., 2017), with LGMD and neuropathic features. Therefore the literature to date suggests that mutations in proteins involved in membrane remodeling and trafficking might act through a shared pathological pathway (Figure 4A), with different diseases likely manifesting due to a different degree of impact on protein activity, regulatory functions, or tissue-specific expression, and which thus requires further experimental validation. To gain further evidence on this shared pathological pathway, we performed a bioinformatic pathway analysis for the genes in Figure 4. We used these genes as input to pull out at least 20 of their interaction partners for each of them to make a dataset (details seen method description). From this dataset, we performed GO enrichment analysis (Figures 4B–D and Supplementary Figure 3) and results showed that most of their interaction partners from these seven proteins are mainly in membrane remodeling process such as clathrin-coated pit/vesicle which is the function of dynamin in clathrin-coated vesicle budding (Mettlen et al., 2009), or membrane ruffle, cellular membrane leading edge from cellular compartment analysis. Other common functions such as pre-/post-synapse, synapse interaction are a common disease-causing mechanism for neuropathy. Similar results were obtained from the biological process analysis (Supplementary Figures 3A,B), we found that the most involved processes were endocytosis, vesicle transport and plasma membrane tubulation. And further confirmed from the molecular function analysis (Supplementary Figure 3C), results showed that the most significant enriched molecular functions were the phosphoinositides metabolizing enzymatic activity and membrane receptor binding or signaling. This confirmed in conjunction to the published experimental evidence (Sauvonnet et al., 2005; Chang-Ileto et al., 2011; Taylor et al., 2011; Vazquez-Molina et al., 2018), on which have elaborated above, that those genes listed in Figure 4A shared a common pathway in the membrane remodeling and trafficking pathway mediated by phosphoinositides.

It was recently shown that MTMR2 can rescue MTM1-myopathy, with the short isoform being more effective (Raess et al., 2017). Serotype 9 recombinant AAV vectors encoding MTMR2 were injected into the tibialis anterior muscle of Mtm1-deficient knockout mice, and a therapeutic effect was observed, suggesting strategies aiming at increasing MTMR2 expression levels in skeletal muscle may be beneficial in the treatment of myotubular myopathy (Danièle et al., 2018). This might be due to the highly conserved structures between MTM1 and MTMR2 resulting in phosphatase activity compensation. Earlier it was shown in a mouse model that dynamin1 was critical for endocytic vesicle recycling under the intense stimulus, while dynamin 2 or dynamin 3 expression were able to partially or completely rescue the endocytic blockade in DNM1-defective neurons (Raimondi et al., 2011). Thus, these complementary and compensatory functions provide a potential therapeutic strategy for the diseases resulting from the common pathological pathway and suggests that one gene may compensate for the loss of function by restoring the overall balance of enzyme activities. However, whether MTM1 could rescue MTMR2-related CMTB1, remains to be investigated.

In conclusion, the cohort hereby reported further expands the clinical and genetic spectrum of CMT4B1. Our patient cohort from developing countries with limited regular access to the state of the medical care represents the unmitigated natural disease course of CMT4B1. Our results suggest that localization of *MTMR2* mutations in certain protein functional domains could be an important determinant of clinical manifestations. The combination of deep clinical phenotyping and electrophysiological studies will be of continued importance in order to guide genetic investigations in this disease.

## Data Availability

The generated and analyzed datasets (novel pathogenic variants) for this study can be found in the ClinVar database (accession numbers: SCV000965697 to SCV000965700; https://www.ncbi.nlm.nih.gov/clinvar/).

## Ethics Statement

The studies involving human participants were reviewed and approved by Ethical boards in University of Cologne/Germany. Written informed consent to participate in this study was provided by the participants' legal guardian/next of kin. Written informed consent was obtained from the individual(s), and minor(s)' legal guardian/next of kin, for the publication of any potentially identifiable images or data included in this article.

## Author Contributions

HW and SC obtained genomic data and wrote the manuscript. RS, KS, YJ, and MP contributed to the clinical descriptions, clinical tables, and the manuscript writing. HG, BS, YJ, EC, IN, SE, RS, MP, HH, VS, and JZ carried out genetic analysis. AK, EC, KS, GS, NB, SN, MY, AS, FB, TS, HP, VS, YJ, MT, RA, and NM collected clinical data. OO performed the pathway analysis. All authors have been involved in final manuscript approval. SC designed, supervised, and obtained funding for the study.

### Conflict of Interest Statement

The authors declare that the research was conducted in the absence of any commercial or financial relationships that could be construed as a potential conflict of interest.
