# Supplementary material for: Genotype-Phenotype Correlations in Charcot-Marie-Tooth Disease Due to MTMR2 Mutations and Implications in Membrane Trafficking
Source: Front Neurosci. 2019 Oct 14;13:974. doi: 10.3389/fnins.2019.00974 (PMC6807680; doi:10.3389/fnins.2019.00974)
Supplement: Supplementary file 1 [file Data_Sheet_1.pdf]

Supplementary materials

Clinical Description

Family 1 Patient II.1

Patient II.1 from family 1 was an 18-year-old male who showed an age appropriate development in his first three years of life. As a first sign of the disease rapid fatigue was noticed. He then began developing bilateral symmetric limb weakness, which progressed slowly to include distal muscles groups and extended to proximal muscle groups in the course of the disease. Between the ages of four to seven years, he was still able to run with other children. In the seventh year, he developed severe walking difficulties. He underwent surgery twice for pes cavus foot deformities at eight years of age. During follow-up in his teenage years, he developed muscle atrophy of all four limbs, facial weakness, dysphagia, speech impairment, left arytenoid subluxation and vocal cord paralysis, mild pectus carinatum, adenoid vegetation with mild nasopharyngeal obstruction and snoring, and a myopic refractive error (-6.00 diopters) (Figure 1A). He had been able to walk with support until the age of 17 years. Since then he was bound to a wheelchair and was not able to close his eyes completely. On the latest follow-up visit with 18 years of age, his somatic development in height and weight was below the 3rd percentile (161.3 cm, 52.5 kg) (Neyzi et al., 2015). The muscle strength of the upper extremities was 3/5 for the proximal muscles and 2/5 for the distal muscles. The muscle strength of the lower extremities was 0/5 for the proximal and distal muscles according to the Medical Research Council (MRC) scale (Medical Research Council. 1976). The patient died at the age of 18.5 years due to respiratory failure.

Family 1 Patient II.2

Patient II.2 from family 1 is a 18-year-old female who also first presented with rapid fatigue at the age of four. Walking difficulties developed at the age of eight and she underwent surgery for pes cavus foot deformities at nine years. Muscle atrophy of all four limbs, facial weakness, dysphagia, speech impairment, adenoid vegetation with mild nasopharyngeal obstruction, left arytenoid subluxation and vocal cord paralysis (Video 1), mild pectus carinatum, pes cavus deformity, and myopic refractive error (-4.50 diopters) developed during follow-up (Figure 1B). She has been able to walk with support and is able to completely shut her eyes until now. Her somatic development in height and weight is also below the 3rd percentile (150.5 cm, 45.3 kg) (Neyzi et al., 2015). Proximal muscle strength of upper and lower extremities are 4/5, distal 3/5 according to the MRC scale (Medical Research Council. 1976). At the time of the last examination, at 18 years, she had become wheelchair bound.

Family 1 Patient II.3

Patient II. 3 from family 1 is a 12-year-old male who also exhibited rapid fatigue at the age of four. He developed walking difficulties at the age of seven and underwent surgery for pes cavus foot deformities at eight years. Muscular atrophy of all four limbs, facial weakness, mild pectus carinatum, thoracic kyphosis, and speech impairment developed during follow-up (Figure 1C). Transnasal flexible fiberoptic laryngoscopy revealed adenoid vegetation with mild nasopharyngeal obstruction and hypoplastic arytenoid cartilages with medial deviation (Video 2). He is able to walk without support and is able to close his eyes completely until now (Video 3). At age of 12, his somatic development in height and weight is like his siblings below the 3rd percentile (135.5 cm, 29.2 kg) (Neyzi et al., 2015). The muscle strength of the upper extremities was 5/5 for the proximal muscles and 4/5 for the distal muscles. The muscle strength of the lower extremities was 5/5 for the proximal muscles. Distal muscle strength of the lower extremities was 4/5 for the left muscles, and 3/5 for the right muscles according to the MRC scale (Medical Research Council. 1976).

Family 2 Patient II.1

Patient II.1 from family 2 is a 29-year-old female with normal motor development till the age of 7 years who also exhibited rapid fatigue at the age of seven. She developed walking difficulties at the age of ten and presented with pes cavus foot deformities. On the latest follow-up at the age of 15 years, she showed muscular atrophy of all four limbs, facial weakness and speech impairment, mild pectus carinatum and thoracic kyphosis. She had been able to walk with support till the age of 15 years but since then, she is wheelchair-bound. The muscle strength of the upper extremities was 3/5 for the proximal muscles and 0/5 for the distal muscles. The muscle strength of the lower extremities was 1/5 for the proximal muscles. Distal muscle strength of the lower extremities was 0/5 for the left muscles and 0/5 for the right muscles. Progressive bulbar muscles weakness induced severe dysphonia and high frequency of voice since her 18th year which has developed to dysphagia two years later. No refractory errors or any visual loss have been reported. She has frequent respiratory infections since the age of 15. Her height and weight were reported to be below the 3rd percentile.

Evaluation of the right median sensory nerve showed reduced amplitude (8.5 µV) and decreased conduction velocity (1st digit-2nd digit 33m/s). The right ulnar sensory nerve showed reduced amplitude (9.6 µV) and decreased conduction velocity (wrist-5th digit 34 m/s). All motor nerve CMAPs were absent. Right sural SNAP had low amplitude and reduced velocity.

Needle evaluation of the right biceps and the right triceps muscles showed increased insertional activity, increased motor unit amplitude, increased motor unit duration, widespread polyphasic potentials, diminished recruitment, and very decreased interference pattern. All remaining muscles

showed no evidence of electrical instability. In conclusion this was interpreted as severe chronic hereditary motor neuropathy with axonal feature compatible with hereditary axonal motor neuropathy.

Family 2 Patient II.3

Patient II.3 from family 2 is a 26-year-old female (Figure 1D, Supplementary Figure S1A) with motor developmental delay in whom the first sign of the disease was early fatigue. She developed bilateral symmetric limb weakness, which progressed slowly to involve distal muscles groups and further extended to bulbar muscle groups in the course of the disease. Between the ages of 18 months to six years, she was still able to run with other children, although requiring support from brace (orthotic) shoes. At the age of 10 years, she developed walking difficulties and has been wheelchair-bound since the age of 15 years. She has lost her vision due to bilateral glaucoma at the age of 14 years. During follow-up at the age of 15 years, she displayed muscle atrophy of all four limbs, facial weakness especially on masticatory muscles that induced inability to close her mouth and masticate without hand force. Muscle strength of the upper extremities was 2/5 for the proximal muscles and 0/5 for the distal muscles. The muscle strength of the lower extremities was 1/5 for the proximal and 0/5 distal muscles. Muscles atrophy has led to a number of skeletal deformities including club feet and clenched hands and she has pes cavus foot deformities. . Her height and weight are below the 3rd percentile.

Investigation of the right sural sensory nerve showed reduced amplitude (3.9 µV). All other sensory nerves SNAPs and motor nerve CMAPs were absent.

Needle evaluation of the right biceps and the right triceps muscle showed increased motor unit amplitude, increased motor unit duration, widespread polyphasic potentials, diminished recruitment, and very decreased interference pattern. All remaining muscles were electrically silent. These findings are consistent with severe chronic hereditary motor neuropathy.

Family 2 Patient II.4

Patient II.4 from family 2 is a 24-year-old male (Supplementary Figure S1B) with normal motor development till the age of 6 years. First symptoms after the age of six years were rapid fatigue. Walking difficulties developed shortly after. Seizures were also documented in his medical history till the age of 10 years of age. At the time of the last examination at the age of 24 years, he walked without support but his gait was abnormal because of his dropped feet. Muscle atrophy of the distal part of lower limbs and pes cavus deformity was present. Bulbar muscle weakness was less severe than in his siblings, he presented moderate dysphonia and speech impairment, no dysphagia and no respiratory difficulty. No refractive error developed during follow-up. He was able to completely shut his eyes. Proximal muscle strength of upper and lower extremities were 3/5 and 2/5 MRC, respectively, and distal muscle strength of upper and lower limbs were 0/5 MRC. Height and weight were normal.

All sensory SNAPs (except for the right sural) and all tested motor nerves CMAPs were absent in the electrophysiological examinations. Proximal muscles showed in needle EMG examination severe chronic neurogenic pattern and distal ones were electrically silent. These findings are consistent with a diagnosis of a severe hereditary axonal type motor neuropathy.

Whole Exome Sequencing and Data Analysis

For family 1 and 2, genomic DNA was extracted from blood with the QIAamp DNA blood mini kit (Qiagen, Germany) and underwent WES on an Illumina HiSeq 4000 sequencing platform (Illumina, USA), using the SureSelect Human All Exon V6 (Agilent, USA) enrichment kit according to the manufacturer's best-practice protocol.

For Family 1, data analysis was performed with the Varbank V.2.18 exome pipeline of the Cologne Centre for Genomics (https://varbank.ccg.uni-koeln.de/), including alignment against the human reference genome hg19 and variant calling. Sequencing data were filtered for rare (minor allele frequency <0.1%), homozygous variants (allele read frequency of 75%–100%) in accordance with the expected autosomal recessive mode of inheritance with the consanguineous background (runs of homozygostity (ROH) > 200). The mean coverage was 91%, 10x coverage for 96,6% and 30x coverage for 87,3% of target sequences.

For family 2, WES was carried out by Novogene (Hong Kong), sequencing reads were mapped to the human reference genome hg19 using the Burrows-Wheeler Aligner (BWA V.0.7.8-r455). Indels and single nucleotide polymorphisms (SNPs) were detected using GATK v3.1 with standard filtering (read depth ≥10% and quality score ≥30) and annotated using ANNOVAR. Mean coverage was 40 fold; 95.9% at 10X coverage, and 59.3% at 30X coverage. Variants affecting protein sequence or splice sites were considered and filtered for rare (minor allele frequency <0.1%), autosomal-recessive variants.

For family 3, genomic DNA from samples was extracted from peripheral blood by standard salting-out protocol. Whole exome sequencing was performed on an Ion Proton platform (Macrogen, Korea) using Life Technologies Ion AmpliSeq Exome Kit and Ion Sphere™ Particle (ISP). Data

analysis including alignment, mapping (to human reference sequence GRCh37), variant calling and variant annotations was performed using ion-torrent software (Torrent-Suite V5.0.2, ion-analysis v5.0.7-1, ion-chefupdates v5.0.1, ion-gpuv5.0.0-1, ion-pipeline v5.0.12-1, ion-pluginsv5.0.17, ion-protonupdatesv5.0.2 and ion-torrentr v 0.0-1. Coverage of the exome was between 10 and 30 fold. Data were filtered for rare (minor allele frequency <0.001 in publicly available population databases GME Variome, ExAC , gnomAD, Iranome and an in-house database including frequency data from approximately 965 population-matched WES) homozygous coding (nonsynonymous, splicing, or truncating) variants. Pathogenicity and potential functional effects of the selected variants were assessed using in silico prediction tools (e.g., SIFT, PolyPhen-2 and MutationTaster). Regions of homozygosity (ROH) for the proband were calculated from VCF files using HomozygosityMapper which uses a sliding blocks algorithm, and has been shown to accurately detect ROH >1.5 Mb (Seelow et al., 2012).

For family 4, WES was performed as described elsewhere (Mencacci et al., 2016). Nextera Rapid Capture Enrichment kit (Illumina) was used according to the manufacturer instructions. Libraries were sequenced in an Illumina HiSeq3000 using a 100-bp paired-end reads protocol. Sequence alignment to the human reference genome (UCSC hg19), and variants call and annotation were performed as described elsewhere (Mencacci et al., 2016) .In total, 99,701,058 unique reads were generated for the proband. After removing all synonymous changes, we filtered single nucleotide variants (SNVs) and indels, only considering exonic and donor/acceptor splicing variants. In accordance with the pedigree and phenotype, priority was given to rare variants [<0.01% in public databases, including 1000 Genomes project, NHLBI Exome Variant Server, Complete Genomics 69, and Exome Aggregation Consortium (ExAC v0.2)] that were fitting a recessive (homozygous or compound heterozygous) or a *de novo* model, and/or variants in genes previously linked to developmental delay, weakness and other neurological disorders.

For all families, filtering was performed according to the expected autosomal-recessive rare variants as described earlier by stringent filtering for variant quality, polymorphisms and deep intronic variants and evaluated for genomic localization, HGNC gene ID, RefSeq ID, variant on cDNA and protein level, ACMG classification, ClinVar annotation, allele frequency according to gnomAD - and GME for patients of middle eastern descent – as well as bioinformatic scores (MutPred, SIFT, PPH, PhyloP) (Richards et al., 2015). Only the observed MTMR2 variants were considered as pathogenic for these phenotypes according to ACMG 2015 criteria.

Supplementary Figure S1 Additional photos from family 2 patients

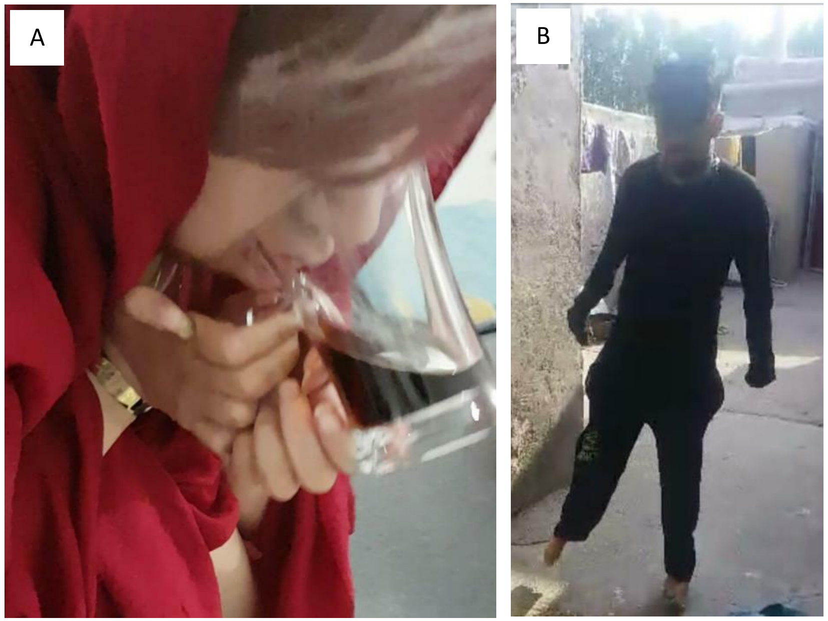

Family 2 Patient II.3 (A) from family 2 showing clenched hands and Patient II. 4 (B) walked without support but his gait is abnormal because of his dropped feet

Supplementary Figure S2 Electromyographic findings of family 2 patients

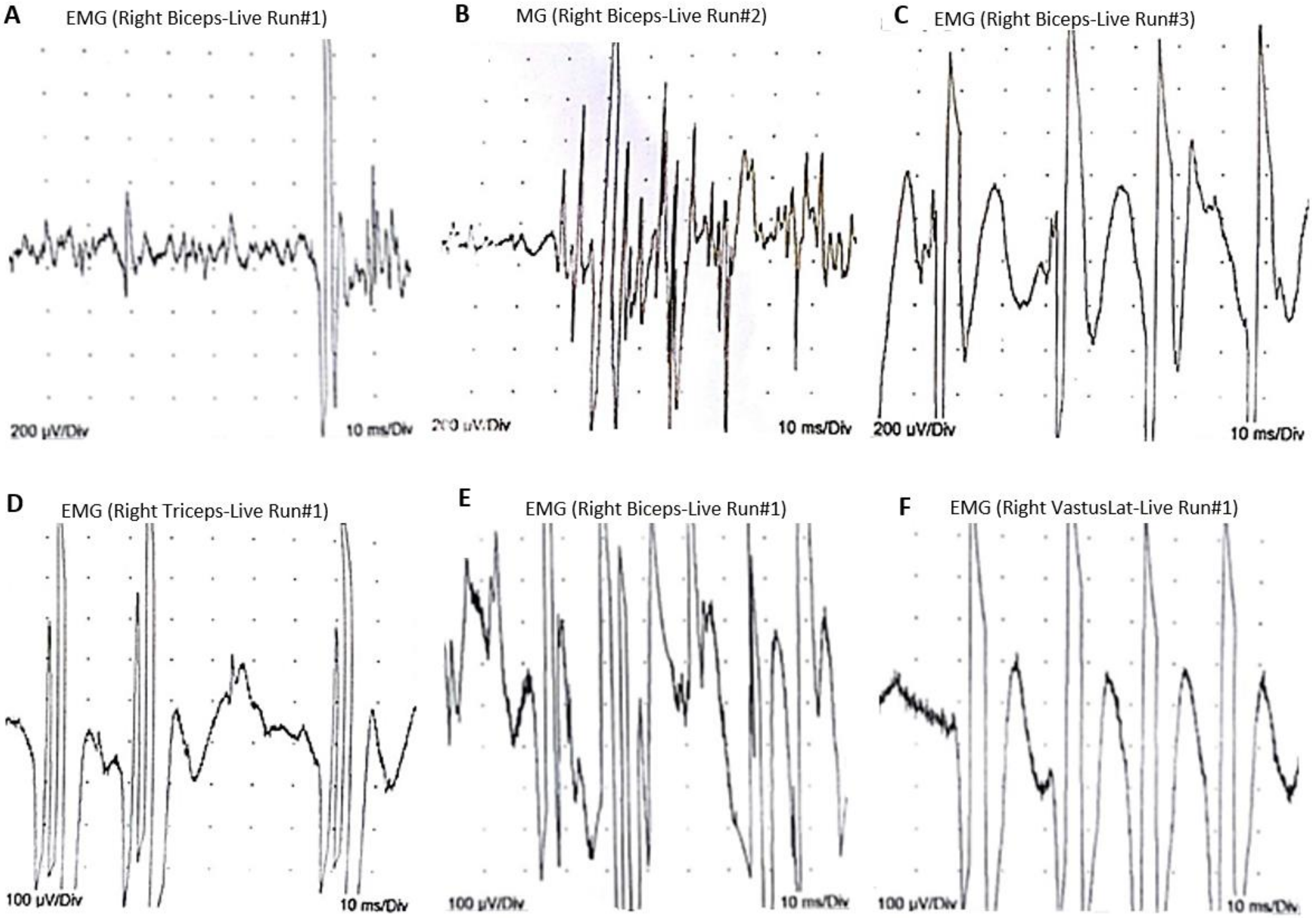

(A-D) Electromyographic findings of patient II.1 from family 2. Needle evaluation of the right biceps and the right triceps muscles showed increased insertional activity, increased motor unit amplitude, increased motor unit duration, widespread polyphasic potentials, diminished recruitment, and very decreased interference pattern. **(E) EMG of patient II.3, family 2.** Needle evaluation of the right biceps muscle showed increased motor unit amplitude, increased motor unit duration, widespread polyphasic potentials, diminished recruitment, and very decreased interference pattern. **(F) EMG of patient II.4, family 2.** Proximal muscles showed in needle examination severe chronic neurogenic pattern, distal ones were electrically silent.

Video 1. Adenoid vegetation with mild nasopharyngeal obstruction, left arytenoid subluxation and left vocal cord paralysis as observed in patient family 1 II.2 by transnasal flexible fiberoptic laryngoscopy.

Video 2. Adenoid vegetation with mild nasopharyngeal obstruction and hypoplastic arytenoid cartilages with medial deviation as observed in patient family 1 II.3 by transnasal flexible fiberoptic laryngoscopy.

Video 3. Although patient family 1 II.3 appears to walk without support, he has difficulty walking due to the weakness of distal lower limb muscles.

Video 4. Adenoid vegetation with mild nasopharyngeal obstruction, left arytenoid subluxation and left vocal cord paralysis as observed in patient family 3 II.2 by transnasal flexible fiberoptic laryngoscopy.

Supplementary Figure S3 The pathway analysis for membrane remodeling genes

A.

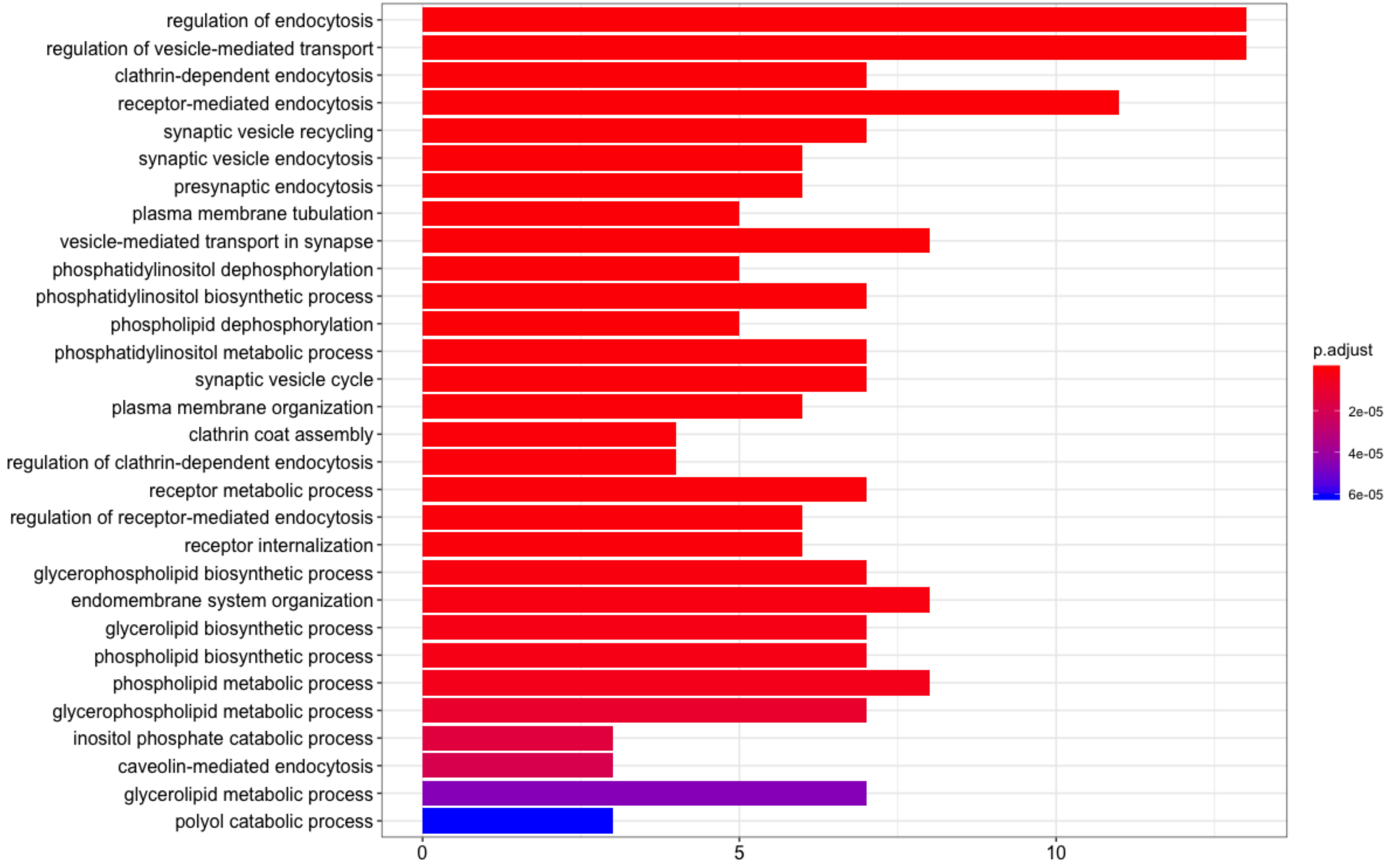

B.

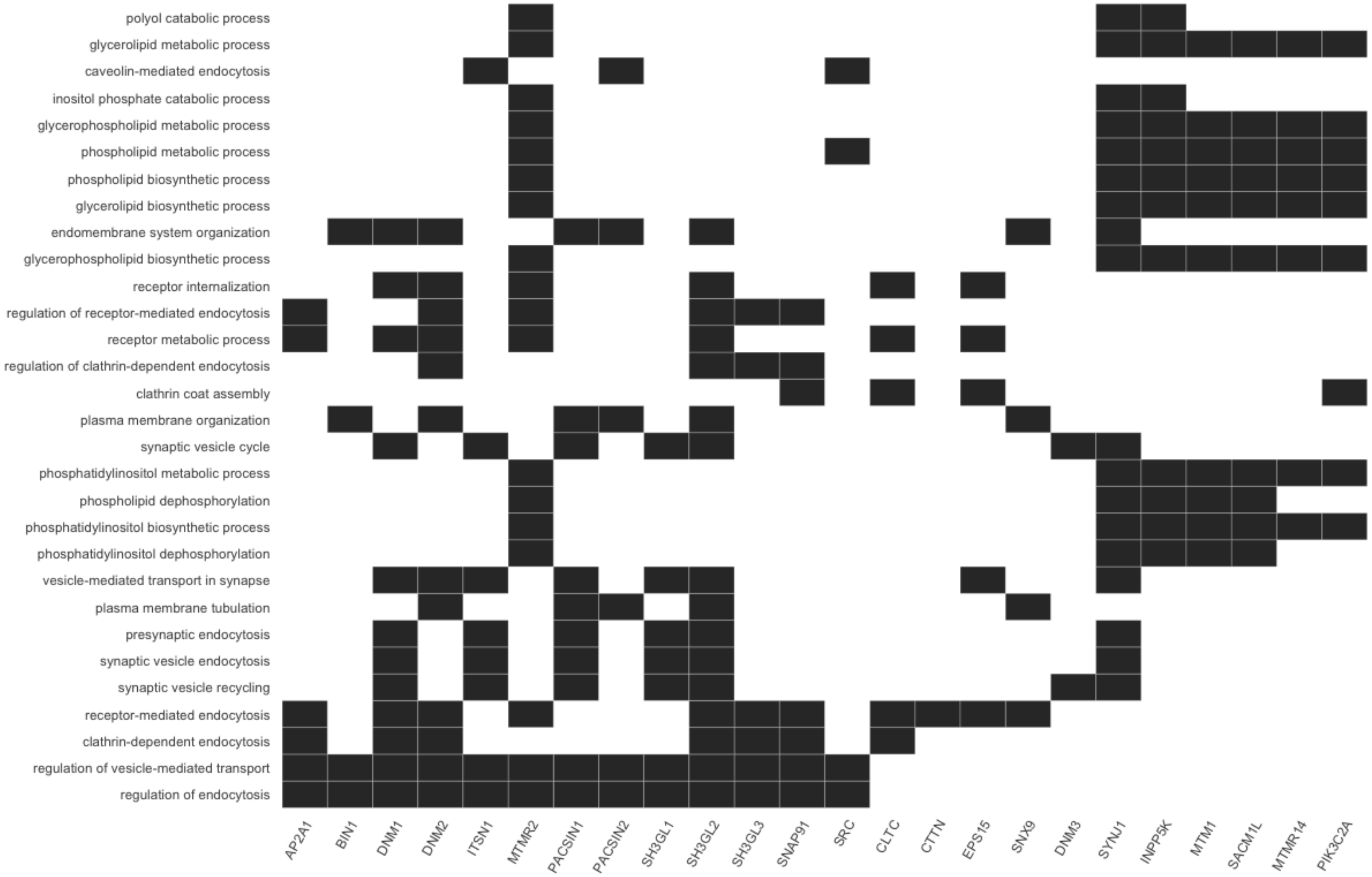
polyol catabolic process
glycerolipid metabolic process
caveolin-mediated endocytosis
inositol phosphate catabolic process
glycerophospholipid metabolic process
phospholipid metabolic process
phospholipid biosynthetic process
glycerolipid biosynthetic process
endomembrane system organization
glycerophospholipid biosynthetic process
receptor internalization
regulation of receptor-mediated endocytosis
receptor metabolic process
regulation of clathrin-dependent endocytosis
clathrin coat assembly
plasma membrane organization
synaptic vesicle cycle
phosphatidylinositol metabolic process
phospholipid dephosphorylation
phosphatidylinositol biosynthetic process
phosphatidylinositol dephosphorylation
vesicle-mediated transport in synapse
plasma membrane tubulation
presynaptic endocytosis
synaptic vesicle endocytosis
synaptic vesicle recycling
receptor-mediated endocytosis
clathrin-dependent endocytosis
regulation of vesicle-mediated transport
regulation of endocytosis
AP2A1
BIN1
DNM1
DNM2
ITSN1
MTMR2
PACSIN1
PACSIN2
SH3GL1
SH3GL2
SH3GL3
SNAP91
SRC
CLTC
CTTN
EPS15
SNX9
DNM3
SYNJ1
INPP5K
MTM1
SACM1L
MTMR14
PIK3C2A

C.

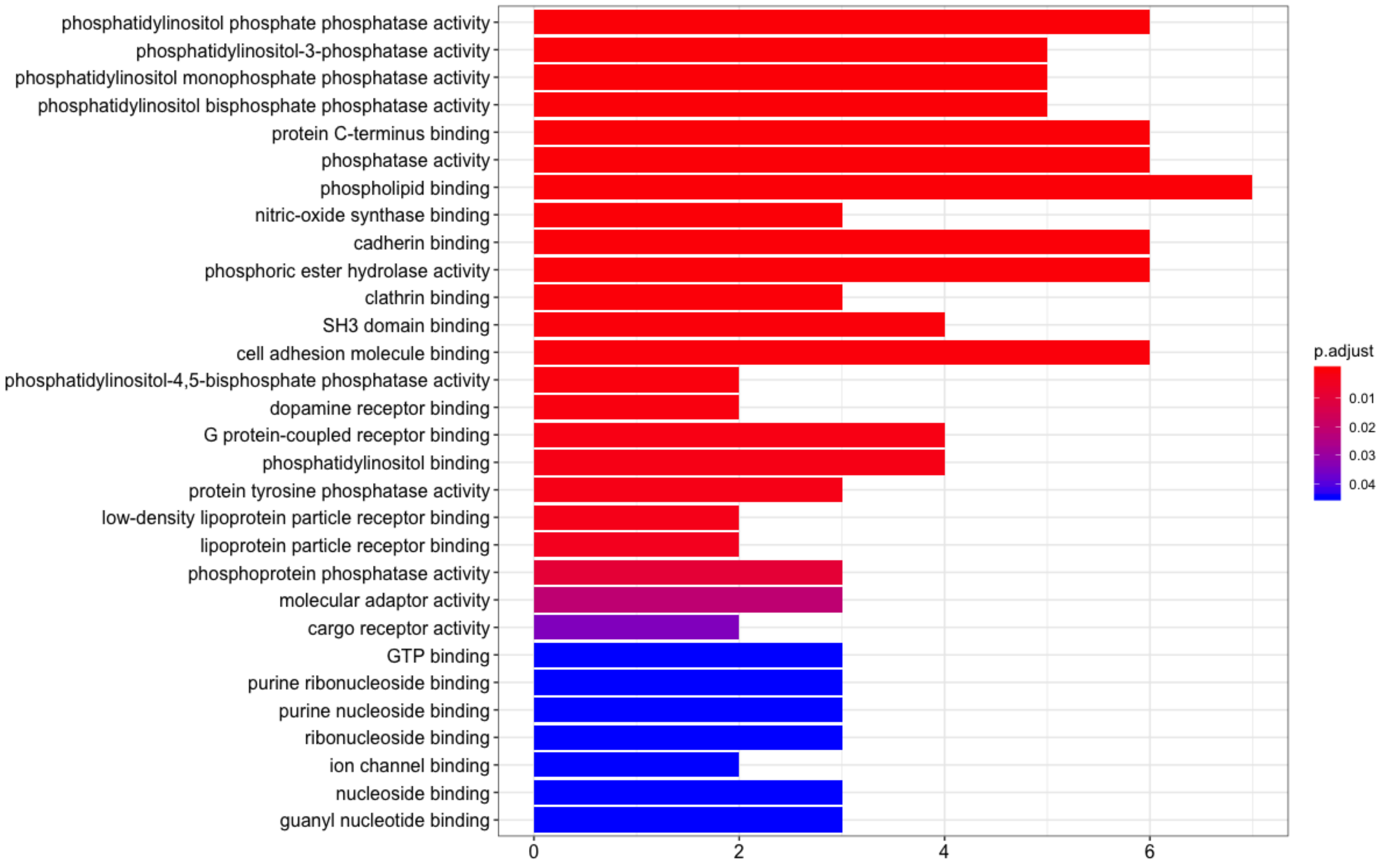

phosphatidylinositol phosphate phosphatase activity
phosphatidylinositol-3-phosphatase activity
phosphatidylinositol monophosphate phosphatase activity
phosphatidylinositol bisphosphate phosphatase activity
protein C-terminus binding
phosphatase activity
phospholipid binding
nitric-oxide synthase binding
cadherin binding
phosphoric ester hydrolase activity
clathrin binding
SH3 domain binding
cell adhesion molecule binding
phosphatidylinositol-4,5-bisphosphate phosphatase activity
dopamine receptor binding
G protein-coupled receptor binding
phosphatidylinositol binding
protein tyrosine phosphatase activity
low-density lipoprotein particle receptor binding
lipoprotein particle receptor binding
phosphoprotein phosphatase activity
molecular adaptor activity
cargo receptor activity
GTP binding
purine ribonucleoside binding
purine nucleoside binding
ribonucleoside binding
ion channel binding
nucleoside binding
guanyl nucleotide binding
0
2
4
6
p.adjust
0.01
0.02
0.03
0.04

D.

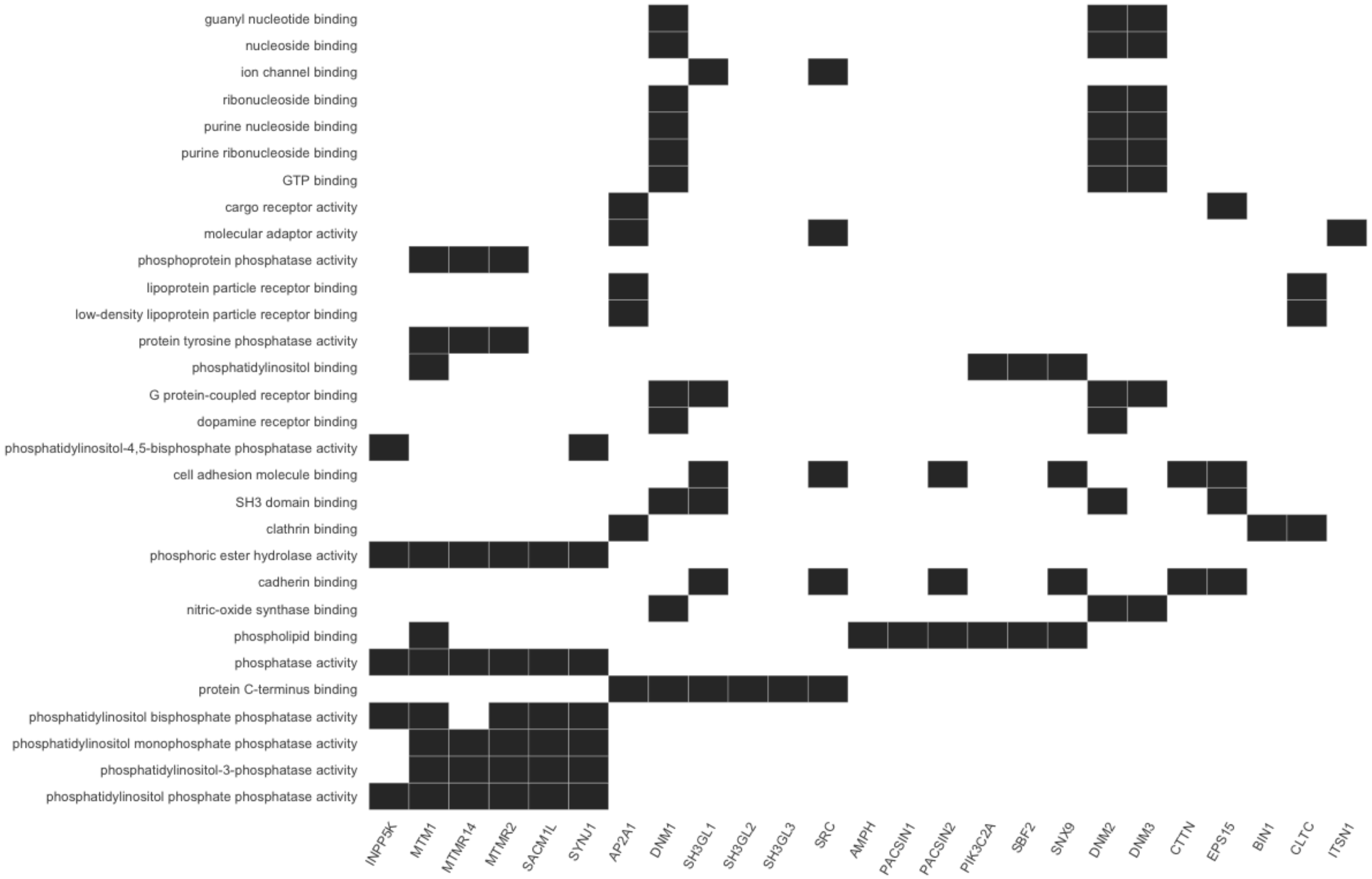

The over-representation analysis has been performed for Gene Ontology (GO), (A-B) Biological Process (BP), (C-D) Molecular Function (MF) classes via R- cluster profiler. Hypergeometric distribution were used to calculate the p-values. False discovery rates were calculated via the

Benjamini-Hochberg method. A total of 27 genes (7 original genes in addition to 20 interactors with the highest score in the interactome analysis) were used for the over-representation analysis. (A) The gene counts enriched for the most significant 30 GO:BP terms are shown in the bar plot. The colors represent the FDR adjusted p-values for each term. *Regulation of endocytosis* and *regulation of vesicle-mediated transport* terms found to be the most significant GO:BP terms with $1.9E^{-14}$,$2.4E10^{-11}$ FDR adjusted p values respectively. (B) The heat plots shows the genes which are over-represented for those GO:BP terms. A total of 13 out of 27 given sets of genes are over-represented including *MTMR2* in the *regulation of endocytosis* and *regulation of vesicle-mediated transport* terms. (C) The gene counts and FDR adjusted p-values for the most significant 30 GO:MF terms are represented in the bar plot. *Phosphatidylinositol phosphate phosphatase activity* GO:MF term which represents the catalysis of the removal of the 4-phosphate group of a phosphatidylinositol phosphate found to be the most significant GO:MF term with the $1.5E^{-10}$ p-value. (D) The genes which are enriched for GO:MF terms are shown in the heat plot.

Supplementary Table Legends

Supplementary Table 1: The details of the interactome analysis are shown in the Edge Table. The interaction nodes and interaction types (protein-protein; protein chemical; chemical-chemical) as well as their confidence scores are given in the table.

Supplementary Table 2: The details of String of StringDB enrichment analysis are given in the String Table. The category of enrichment, description of the enriched database/term/publication and enriched genes as well as their FDR adjusted p-values and public IDs are given in the table.

Supplementary Table 3: Details of GO:CC over-representation analysis results are given in the GO:CC ORA table. The whole list of GO terms which are found to be significant in the analysis, p-values before and after FDR adjustment and the gene ratios as well as over-represented genes are shown in the table.

Supplementary Table 4: Details of GO:BP over-representation analysis results are given in the GO:BP ORA table. The whole list of GO terms which are found to be significant in the analysis, p-values before and after FDR adjustment and the gene ratios as well as over-represented genes are shown in the table.

Supplementary Table 5: Details of GO:MF over-representation analysis results are given in the GO:BP ORA table. The whole list of GO terms which are found to be significant in the analysis, p-values before and after FDR adjustment and the gene ratios as well as over-represented genes are shown in the table.

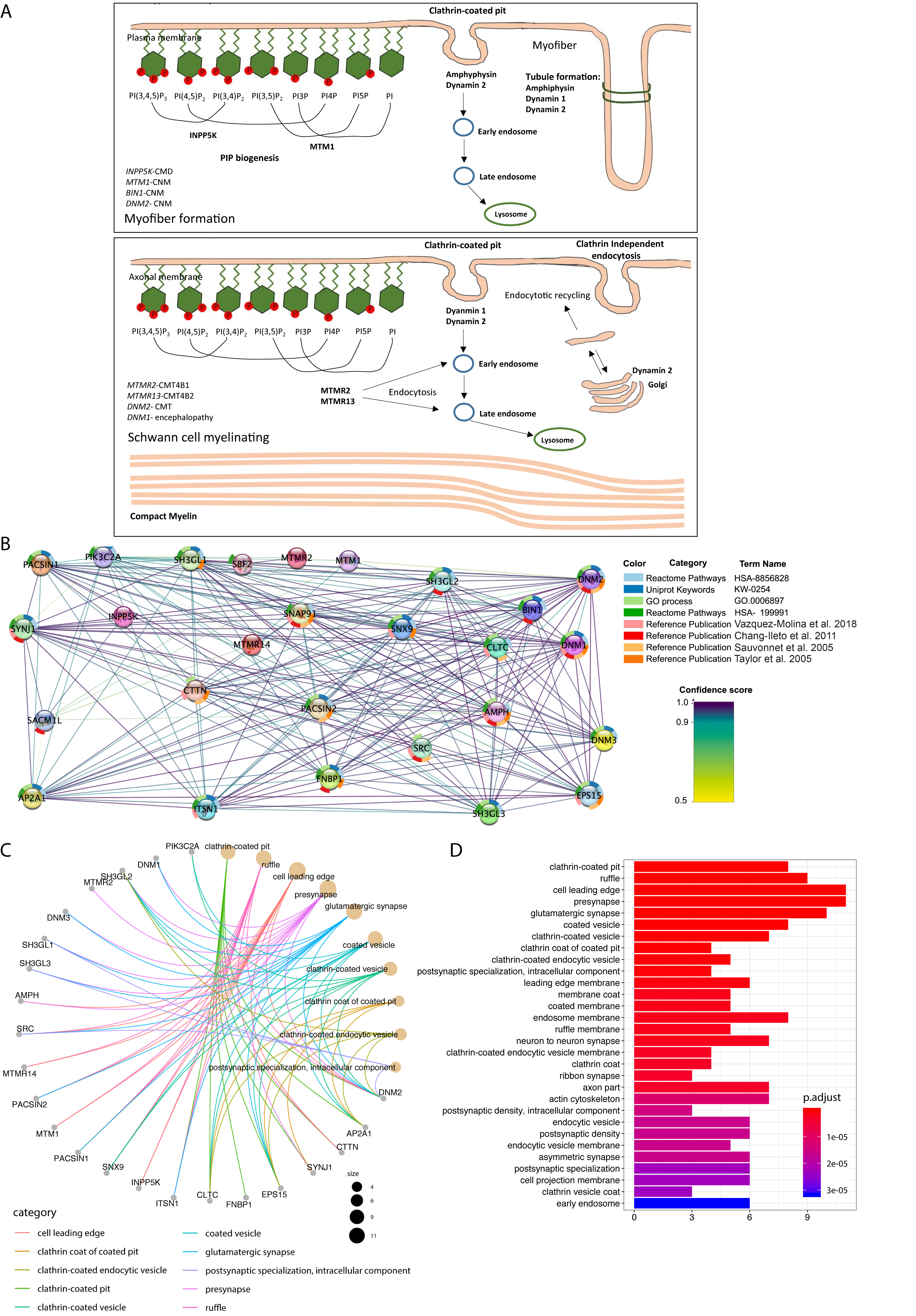
A
Clathrin-coated pit
Plasma membrane
Myofiber
PI(3,4,5)P3 PI(4,5)P2 PI(3,4)P2 PI(3,5)P2 PI3P PI4P PI5P PI
Amphyphysin
Dynamin 2
Tubule formation:
Amphiphysin
Dynamin 1
Dynamin 2
Early endosome
Late endosome
Lysosome
INPP5K
MTM1
PIP biogenesis
INPP5K-CMD
MTM1-CNM
BIN1-CNM
DNM2- CNM
Myofiber formation
Clathrin-coated pit
Clathrin Independent endocytosis
Axonal membrane
Endocytotic recycling
Dyanmin 1
Dynamin 2
Early endosome
Late endosome
Lysosome
Dynamin 2
Golgi
MTMR2
MTMR13
Endocytosis
MTMR2-CMT4B1
MTMR13-CMT4B2
DNM2- CMT
DNM1- encephalopathy
Schwann cell myelinating
Compact Myelin
B
PACSIN1
PIK3C2A
SH3GL1
SBF2
MTMR2
MTM1
SH3GL2
DNM2
INPP5K
SNAP91
BIN1
SYNJ1
SNX9
MTMR14
CLTC
DNM1
CTTN
SACM1L
PACSIN2
AMPH
DNM3
SRC
FNBP1
AP2A1
ITSN1
SH3GL3
EPS15
Color Category Term Name
Reactome Pathways HSA-8856828
Uniprot Keywords KW-0254
GO process GO.0006897
Reactome Pathways HSA- 199991
Reference Publication Vazquez-Molina et al. 2018
Reference Publication Chang-Ileto et al. 2011
Reference Publication Sauvonnet et al. 2005
Reference Publication Taylor et al. 2005
Confidence score
1.0
0.9
0.5
C
PIK3C2A
clathrin-coated pit
DNM1
ruffle
SH3GL2
MTMR2
cell leading edge
presynapse
glutamatergic synapse
DNM3
coated vesicle
SH3GL1
SH3GL3
clathrin-coated vesicle
AMPH
clathrin coat of coated pit
SRC
clathrin-coated endocytic vesicle
MTMR14
postsynaptic specialization, intracellular component
DNM2
PACSIN2
MTM1
AP2A1
CTTN
PACSIN1
SNX9
SYNJ1
INPP5K
CLTC
EPS15
ITSN1
FNBP1
size
4
6
9
11
category
cell leading edge
clathrin coat of coated pit
clathrin-coated endocytic vesicle
clathrin-coated pit
clathrin-coated vesicle
coated vesicle
glutamatergic synapse
postsynaptic specialization, intracellular component
presynapse
ruffle
D
clathrin-coated pit
ruffle
cell leading edge
presynapse
glutamatergic synapse
coated vesicle
clathrin-coated vesicle
clathrin coat of coated pit
clathrin-coated endocytic vesicle
postsynaptic specialization, intracellular component
leading edge membrane
membrane coat
coated membrane
endosome membrane
ruffle membrane
neuron to neuron synapse
clathrin-coated endocytic vesicle membrane
clathrin coat
ribbon synapse
axon part
actin cytoskeleton
postsynaptic density, intracellular component
endocytic vesicle
postsynaptic density
endocytic vesicle membrane
asymmetric synapse
postsynaptic specialization
cell projection membrane
clathrin vesicle coat
early endosome
0
3
6
9
p.adjust
1e-05
2e-05
3e-05
